# Percutaneous Management of Pyogenic Hepatic Abscesses

**DOI:** 10.1155/1990/28345

**Published:** 1990

**Authors:** Roland Andersson, Lillemor Forsberg, Esbjörn Hederstrom, Peter Hochbergs, Stig Bengmark

**Affiliations:** 1 Department of Surgery Lund University Sweden; 2 Department of Roentgenology Lund University Sweden; 3 Department of Surgery Lund University Lund S-221 85 Sweden

## Abstract

Twelve patients (9 men, 3 women) with a mean age of 65 (54–78) years, with pyogenic hepatic abscesses were managed by percutaneous drainage between 1979 and 1987. Biliary origin was most common (4 patients), followed by hepatic abscesses as a late postoperative complication (seen in 3 patients) and hepatic abscesses occurring in association with acute appendicitis (2 patients). The origin was unknown in 3 patients. Diagnosis was reached by computed tomography or ultrasonography with a diagnostic delay of in mean 11 days. Seventeen abscesses were found among the 12 patients. The median abscess size (maximal diameter) was 7 (1–12) cm. Nine patients were treated with percutaneous drainage with an indwelling catheter within the abscess cavity for up to 3 weeks, while 3 patients were managed with percutaneous puncture and aspiration alone. The most commonly isolated organism from the drained hepatic abscess was E.coli. The course following percutaneous treatment was uneventful, without mortality and recurrence of the hepatic abscess during follow-up. One patient required surgical drainage of an additional hepatic abscess.

Percutaneous drainage of hepatic abscesses, independent of origin, thus seems as a safe and reliable method, which should be considered as the treatment of choice if facilities and knowledge of percutaneous management are provided.


*HPB Surgery*, 1990, Vol. 2, pp. 185–188
Reprints available directly from the publisher
Photocopying permitted by license only

© 1990 Harwood Academic Publishers GmbH
Printed in the United Kingdom

### PERCUTANEOUS MANAGEMENT OF PYOGENIC HEPATIC ABSCESSES

ROLAND ANDERSSON, LILLEMOR FORSBERG*, ESBJÖRN HEDERSTROM*, PETER HOCHBERGS* and STIG BENGMARK
*Departments of Surgery and Roentgenology*, Lund University, Sweden*

Twelve patients (9 men, 3 women) with a mean age of 65 (54–78) years, with pyogenic hepatic abscesses were managed by percutaneous drainage between 1979 and 1987. Biliary origin was most common (4 patients), followed by hepatic abscesses as a late postoperative complication (seen in 3 patients) and hepatic abscesses occurring in association with acute appendicitis (2 patients). The origin was unknown in 3 patients. Diagnosis was reached by computed tomography or ultrasonography with a diagnostic delay of in mean 11 days. Seventeen abscesses were found among the 12 patients. The median abscess size (maximal diameter) was 7 (1–12) cm. Nine patients were treated with percutaneous drainage with an indwelling catheter within the abscess cavity for up to 3 weeks, while 3 patients were managed with percutaneous puncture and aspiration alone. The most commonly isolated organism from the drained hepatic abscess was E.coli. The course following percutaneous treatment was uneventful, without mortality and recurrence of the hepatic abscess during follow-up. One patient required surgical drainage of an additional hepatic abscess.

Percutaneous drainage of hepatic abscesses, independent of origin, thus seems as a safe and reliable method, which should be considered as the treatment of choice if facilities and knowledge of percutaneous management are provided.

KEY WORDS: Liver, abscess, drainage, percutaneous, ultrasonography, computed tomography

### INTRODUCTION

The outcome of patients with hepatic abscesses has previously been poor. These critically ill patients have in general a long period of illness before admission and also a long delay until correct diagnosis is reached. Pyogenic hepatic abscesses, especially when multiple, have been associated with mortality rates at or above 25%, despite improvements in diagnosis, antibiotics and surgical therapy[1-4]. However, during the last decade a marked decrease in mortality has been noted in association with the introduction of new imaging techniques, such as computed tomography and ultrasonography, leading to a decrease in diagnostic delay and making percutaneous drainage possible [5-9]. The present study reviews our experience of percutaneous management of hepatic abscesses with the guidance of computed tomography or ultrasonography.

---

Correspondence to: Roland Andersson, M.D. Department of Surgery, Lund University, S-221 85 LUND, Sweden

### PATIENTS AND METHODS

During the period 1979 to 1987, 12 patients (9 men, 3 women; mean age 65(54–78) years) with pyogenic hepatic abscesses were treated with percutaneous drainage. During the same period of time, only one patient was assigned to primary surgery of an hepatic abscess, due to technical reasons with an interpositioned colonic loop not rendering the abscess available for percutaneous management. The length of illness before admission among patients treated percutaneously was in mean 25 days. Symptoms were frequently obscure, merely being signs of sepsis of unknown origin or malignancy. The hepatic abscesses occurred as late postoperative complications in 3 patients (following gastric surgery; perforated ulcer in 2, gastric resection in one), while the etiology among the remaining patients was biliary in 4, appendicitis in 2 and unknown in 3 patients. The diagnosis was reached by computed tomography in 4, ultrasonography in 5 or both of these investigations in 3 patients. The delay from admission until the diagnosis was established was 11 (mean; range 1–21) days. Seventeen hepatic abscesses were diagnosed among the 12 patients: 9 patients had one abscess, 3 patients had 2 and one patient had 3 hepatic abscesses respectively. The median size of the abscess (maximal diameter) was 7 (range 1–12) cm. Nine patients had abscesses within the right liver lobe, one in the left and 2 patients had abscesses in both the right and left liver lobes.

*Percutaneous management*

When a suspected hepatic abscess was found on CT or ultrasonography, the diagnosis was verified by percutaneous aspiration, then yielding pus. Specimens were taken for aerobic and anaerobic bacterial culture, as well as for cytology. If a percutaneous catheter drainage was considered feasible (9 patients), the shortest way from the skin to the hepatic abscess, using a transperitoneal route, was chosen. The puncture site was then marked out and local anaesthetics was administered. The hepatic abscess was punctured with a polyetylene catheter sheathed needle (trocar catheter drainage set 7-F, MEDA, Sweden, or Ring-McLean sump drainage set 12-F, 16-F, William Cook, Denmark) under guidance of ultrasound alone in 4 patients and ultrasound and fluoroscopy in combination in 2 patients. Computed tomography was used in 2 patients and fluoroscopy alone in one patient. The sheath was supplied with several sideholes at the tip to facilitate drainage. With the aid of a guidewire the catheter was coiled within the abscess cavity in order to reduce the risk of displacement. If needed, the catheter was flushed daily with saline. Three patients had two catheters inserted. The catheters were removed when drainage had ceased, the patients' condition had improved and they were stable, and when control CT/ultrasonography showed resolution. In 3 patients, the contents within the abscess was aspirated percutaneously and no catheters were inserted. All patients received antibiotics.

### RESULTS

Bacterial cultures from the drained hepatic abscess were positive in 75% of instances, with E.coli being the most frequently isolated organism (in 5 patients). Blood cultures did not show any evidence of bacterial growth, except in one patient where enterobacter cloacae was cultured.

In patients with percutaneous catheter drainage, the catheter was left in the abscess cavity for in median 14 days (range 3–21 days).

All patients survived. Following percutaneous drainage, the patients gradually improved. No complications attributable to the percutaneous procedure were seen. One patient, however, where technical reasons contraindicated a repeated percutaneous drainage, required surgical drainage of an additional hepatic abscess.

### DISCUSSION

Despite the infrequency of pyogenic abscesses, they have constituted a major problem with high mortality rates. By the guidance of high resolution imaging techniques it has been possible to perform percutaneous drainage, whereby the patients can be managed without general anaesthesia and surgery. The drainage technique has become safer, especially when using ultrasonic punction devices where the catheter can be seen entering the cavity. In some patients, it can be more convenient to fill the cavity with contrast medium, using ultrasonic guidance and thereafter place the catheter under fluoroscopic control. Although the number of patients treated in this fashion so far is limited, reported mortality rates has been at or close to zero [6–9]. Despite this, it has recently been claimed that surgical drainage remains the standard [10] and percutaneous drainage could be reserved for high risk patients [2–6]. As mortality and morbidity rates following surgical drainage still are considerable and in the light of the good results reported following percutaneous management, both in terms of a low morbidity rate and a low recurrence rate, percutaneous treatment of hepatic abscesses, if available, should be thought of as the treatment of choice, unless the puncture procedure *per se* is contraindicated due to technical reasons. The safety and adequacy of percutaneous drainage of hepatic abscesses is supported by the present study. Furthermore, site of origin of the abscess or the occurrence of multiple abscesses within the liver did not contraindicate and did not influence on outcome.

Thus, percutaneous drainage of hepatic abscesses seems as a safe procedure rendering a favourable outcome, provided that the period of catheter drainage is long enough.

### *References*

1. Conter, R.L., Pitt, H.A., Tompkins, R.K. and Longmire Jr, W.P. (1986) Differentiation of pyogenic from amebic hepatic abscesses. *Surg. Gynec. Obst.*, **162**, 114–120.
2. Miedema, B.W. and Dineen, P. (1984) The diagnosis and treatment of pyogenic liver abscesses. *Ann. Surg.*, **200**, 328–335.
3. Pitt, H.A. and Zuidema, G.D. (1975) Factors influencing mortality in the treatment of pyogenic hepatic abscesses. *Surg. Gynec. Obst.*, **140**, 228–234.
4. Rubin, R.H., Swartz, M.N. and Malt, R. (1974) Hepatic abscess: changes in clinical, bacteriologic and therapeutic aspects. *Am. J. Med.*, **57**, 601–610.
5. Falk, K.A., Angeras, U.J., Friman, V.Z., Gamklou, G.R. and Lukes (1987) Pyogenic liver abscesses: have changes in management improved the outcome? *Acta Chir. Scand.*, **153**, 661–664.
6. Farges, O., Leese, T. and Bismuth, H. (1988) Pyogenic liver abscesses: an improvement in prognosis. *Br. J. Surg.*, **75**, 862--865.
7. Gerzof, S.G., Johnson, W.C., Robbins, A.H. and Nabseth, D.C. (1985) Intrahepatic pyogenic abscesses: treatment by percutaneous drainage. *Am. J. Surg.*, **149**, 487–493.
8. Mandel, S.R., Boyd, D., Jaques, P.F., Mandell, V. and Staab, E.V. (1983) Drainage of hepatic, intraabdominal, and mediastinal abscesses guided by computorized axial tomography. *Am. J. Surg.*, **145**, 120–124.

9. Sheinfeld, A.M., Steiner, A.E., Rivkin, L.B., Dermer, R.H., Shemesh, O.V. and Dolberg, M.S. (1982) Transcutaneous drainage of abscesses of the liver guided by computed tomography scan. *Surg. Gynec. Obsi.*, **155**, 662–666.
10. Klatchko, B.A. and Schwartz, S.I. (1989) Diagnostic and therapeutic approaches to pyogenic abscess of the liver. *Surg. Gynec. Obst.*, **168**, 332–336.

*(Accepted by L. H. Blumgart)*

